# Hemin enhances the cardioprotective effects of mesenchymal stem cell-derived exosomes against infarction via amelioration of cardiomyocyte senescence

**DOI:** 10.1186/s12951-021-01077-y

**Published:** 2021-10-21

**Authors:** Huifeng Zheng, Xiaoting Liang, Qian Han, Zhuang Shao, Yuxiao Zhang, Linli Shi, Yimei Hong, Weifeng Li, Cong Mai, Qingwen Mo, Qingling Fu, Xiaoxue Ma, Fang Lin, Mimi Li, Bei Hu, Xin Li, Yuelin Zhang

**Affiliations:** 1 The Second School of Clinical Medicine, Southern Medical University, Guangzhou, 510515 Guangdong China; 2 Department of Emergency Medicine, Guangdong Provincial People’s Hospital, Guangdong Academy of Medical Sciences, Guangzhou, 510080 Guangdong China; 3 Institute for Regenerative Medicine, Shanghai East Hospital, School of Life Sciences and Technology, Tongji University, Shanghai, 200120 China; 4 Department of Respiratory Medicine, State Key Laboratory of Respiratory Disease, The First Affiliated Hospital of Guangzhou Medical University, Guangzhou Institute of Respiratory Health, Guangzhou, 510120 Guangdong China; 5 Otorhinolaryngology Hospital, The First Affiliated Hospital, Sun Yat-sen University, Guangzhou, 510080 Guangdong China; 6 Research Center for Translational Medicine, Shanghai East Hospital, School of Medicine, Tongji University, Shanghai, 200120 China

**Keywords:** Mesenchymal stem cells, Exosomes, Hemin, Cardiomyocytes, Senescence, Myocardial infarction

## Abstract

**Background:**

Application of mesenchymal stem cell-derived exosomes (MSC-EXO) has emerged as a novel therapeutic strategy for myocardial infarction (MI). Our previous study showed that pretreatment with hemin, a potent heme oxygenase-1 (HO-1) inducer, enhanced the cardioprotective effects of MSCs in a mouse model of MI. This study aimed to investigate the therapeutic effects of EXO derived from hemin-pretreated MSCs (Hemin-MSC-EXO) in MI and explore the potential mechanisms.

**Methods:**

MSC-EXO and Hemin-MSC-EXO were collected and characterized. MSC-EXO and Hemin-MSC-EXO were intramuscularly injected into the peri-infarct region in a mouse model of MI. Heart function of mice was assessed by echocardiography. The mitochondrial morphology of neonatal mice cardiomyocytes (NMCMs) under serum deprivation and hypoxic (SD/H) conditions was examined by Mitotracker staining. The cellular senescence of NMCMs was determined by senescence-associated-β-galactosidase assay. A loss-of-function approach was adopted to determine the role of Hemin-MSC-exosomal-miR-183-5p in the regulation of cardiomyocyte senescence

**Results:**

EXO were successfully isolated from the supernatant of MSCs and Hemin-pretreated MSCs. Compared with MSC-EXO, injection of Hemin-MSC-EXO significantly improved cardiac function and reduced fibrosis. Both MSC-EXO and Hemin-MSC-EXO ameliorated cardiomyocyte senescence and mitochondrial fission in vitro and in vivo, and the latter exhibited better protective effects. MicroRNA sequencing revealed a higher level of miR-183-5p in Hemin-MSC-EXO than in MSC-EXO. MiR-183-5p knockdown partially abrogated the protective effects of Hemin-MSC-EXO in attenuating mitochondrial fission and cellular senescence of cardiomyocytes induced by SD/H. High mobility group box-1 (HMGB1) abundance was lower in Hemin-MSC-EXO-treated than MSC-EXO-treated mouse hearts, and HMGB1 was identified as one of the potential target genes of miR-183-5p. Mechanistically, Hemin-MSC-EXO inhibited SD/H-induced cardiomyocyte senescence partially by delivering miR-183-5p into recipient cardiomyocytes via regulation of the HMGB1/ERK pathway. Furthermore, knockdown of miR-183-5p reduced the Hemin-MSC-EXO-mediated cardioprotective effects in a mouse model of MI.

**Conclusion:**

Our results reveal that Hemin-MSC-EXO are superior to MSC-EXO in treating MI. Exosomal miR-183-5p mediates, at least partially, the cardioprotective effects of Hemin-MSC-EXO by inhibiting cardiomyocyte senescence via regulation of the HMGB1/ERK pathway. This study highlights that MSC-EXO have high translational value in repairing cardiac dysfunction following infarction.

**Graphic abstract:**

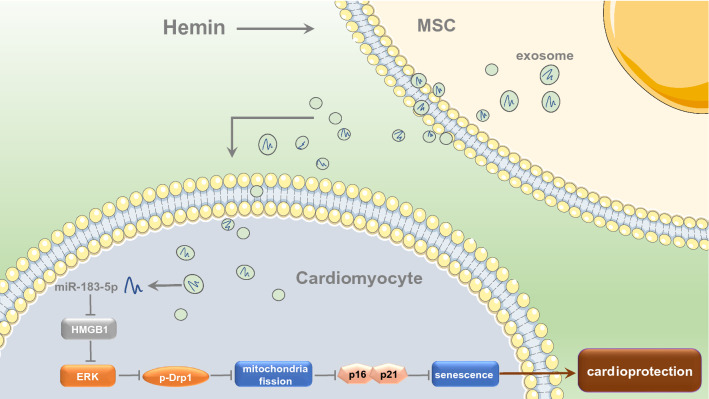

**Supplementary Information:**

The online version contains supplementary material available at 10.1186/s12951-021-01077-y.

## Introduction

Myocardial infarction (MI) is the leading cause of morbidity and mortality worldwide. MI pathologically manifests as cardiomyocyte death due to deficiencies in oxygen and nutrition caused by occlusion of the coronary artery. In addition to traditional necrosis and apoptosis, several new forms of programmed cell death, including pyroptosis and ferroptosis, have been reported to mediate cardiac dysfunction after MI [1–3]. Despite advances in pharmacological and surgical therapy, the clinical outcome for patients with MI remains poor, likely due to comprehensive mechanisms that underlie cardiomyocyte injury [4, 5]. Understanding the potential mechanisms that underlie MI will aid in the development of innovative therapies.

Cellular senescence is a stable state of irreversible cell-cycle arrest characterized by distinctive alterations in the expression of various genes and increased senescence-associated β-galactosidase (SA-β-gal) activity [6, 7]. Furthermore, senescent cells secrete a senescence-associated secretory phenotype (SASP) consisting of various proinflammatory cytokines and growth factors. Although cardiomyocytes are terminally differentiated, senescent cardiomyocytes have been observed in various cardiovascular diseases including MI [7]. Senescent cardiomyocytes display mitochondrial dysfunction, contractile dysfunction and hypertrophic growth, leading to cardiac dysfunction and failure. Furthermore, senescent cardiomyocytes partially impair the function of non-myocytes, including endothelial cells and fibroblasts, via SASP factors, thus accelerating heart injury [8]. Therefore, inhibition of senescent cardiomyocytes in the ischemic heart may be a novel strategy to treat MI. Treatment with the senolytic drug navitoclax has been shown to clear the cardiomyocyte senescence induced by ischemia–reperfusion in mice and significantly improve heart function by attenuating pro-inflammatory and profibrotic SASP, suggesting that clearance of senescent cardiomyocytes can improve heart function following infarction [9]. Nonetheless the precise molecular mechanisms underlying cardiomyocyte senescence in the heart following infarction remain unclear.

Cellular senescence can be induced by various stressors including mitochondrial dysfunction, autophagy dysfunction, oxidative stress and telomere erosion [10–12]. Mitochondria undergo mitochondrial fission, mediated by dynamin-related protein 1 (Drp1) and fission 1 (Fis1), and fusion, regulated by Mitofusin 1 (Mfn1), Mitofusin 2 (Mfn2) and optic atrophy protein 1 (OPA1), to maintain their function. Accumulating evidence has demonstrated that disruption of the balance between mitochondrial fission and fusion mediates cellular senescence [10, 13, 14]. Hypoxic stress-induced Drp1–filamin A interaction has been reported to cause mitochondrial hyperfission, leading to cardiomyocyte senescence in a mouse model of MI [15]. Importantly, pharmacological disruption of the Drp1-filamin A interaction attenuated mitochondrial hyperfission–associated myocardial senescence and improved heart function following infarction. Nonetheless the molecular mechanisms underlying mitochondrial fission-mediated cardiomyocyte senescence induced by ischemia have not been demonstrated.

Over the past decades, mesenchymal stem cell-derived exosomes (MSC-EXO) have shown promising cardioprotective effects against MI [16, 17]. Transplantation of enriched-miR-132-MSC-EXO has been shown to improve heart function in a mouse model of MI by increasing neovascularization in the peri-infarct zone [18]. In addition, transplantation of LncRNA KLF3-AS1-MSC-EXO has been shown to attenuate MI progression by inhibiting cardiomyocyte pyroptosis via regulation of the miR-138-5p/Sirt1 pathway [19]. MSC-EXO exert beneficial effects via bioactive substances that include proteins, mRNAs, LncRNA and miRNAs. Notably, the substances in MSC-EXO are largely influenced by environmental stimuli that alters their biological effect [20, 21]. Thus, optimizing MSC-EXO in vitro to enhance their therapeutic effects is of great importance. Several strategies, including genetic modification, hypoxia preconditioning and pharmacological pretreatment, have been adopted to optimize MSC-EXO and enhance their cardioprotective effects [22, 23]. Our previous study showed that pretreatment with hemin, a potent heme oxygenase-1 (HO-1) inducer, enhanced the cardioprotective effects of MSCs in MI [24]. HO-1, a stress-inducible protein highly expressed in tissues, catalyzes the degradation of heme into biliverdin, free divalent iron, and carbon oxidant. It has been reported that HO-1 exerts multiple cytoprotective actions including anti-oxidative stress, anti-inflammatory and anti-apoptosis effects [25]. Nonetheless whether hemin pretreatment can improve the therapeutic effects of MSC-EXO in MI and the potential underlying mechanisms have not been determined. In this study, we aimed to investigate the therapeutic effects of hemin pretreated-MSC-EXO (Hemin-MSC-EXO) on MI and explore the potential molecular mechanisms.

## Materials and methods

### Cell culture

MSCs were purchased from Cambrex BioScience and cultured as previously described [10]. MSCs were cultured on Dulbecco’s Modified Eagle’s medium (DMEM) supplemented with 10% fetal calf serum (FBS, Life Technologies, 16,000), 5 ng/mL fibroblast growth factor (bFGF, PeProTech, 100-18B), and 10 ng/mL epidermal growth factor (EGF, PeProTech, AF-100-15). MSCs at passage 3–4 were used in the current study. Neonatal mouse cardiomyocytes (NMCMs) were isolated and cultured as described previously [26], 37 °C in a 6-well culture plate containing 2 mL/per well Claycomb Medium (Sigma, 51800).

### MSC-EXO extraction and characterization

MSC-EXO were isolated and characterized as previously reported [27]. Briefly, when MSCs reached 70–80% confluence, medium was replaced by DMEM supplemented with 10% exosome-depleted FBS (dFBS) (Systems Biosciences) and cultured for 48 h. For hemin pretreatment, MSCs were cultured for 24 h under normoxic conditions in complete medium containing 10 µM hemin. After 48 h, supernatant was collected and EXO isolated and purified by anion exchange chromatography. Subsequently, EXO were suspended in PBS and their concentration determined using a bicinchoninic acid (BCA) kit. The particle size of EXO was analyzed using nanoparticle tracking analysis (NTA). Transmission electron microscopy (TEM) and western blotting for CD63, CD81, TGS101 and Alix were used to characterize the collected MSC-EXO and hemin-MSC-EXO.

### Internalization of EXO

MSC-EXO were labeled with Dil (Beyotime) and then co-cultured with NMCMs for 48 h. Next, NMCMs were washed with PBS three times and fixed in 4% paraformaldehyde. The internalized MSC-EXO in NMCMs were evaluated under a fluorescence microscope.

### SA-β-gal assay

The senescence of NMCMs was evaluated by SA-β-gal staining (Beyotime, C0602). Briefly, NMCMs cultured in 6-well plates were treated with PBS, MSC-EXO (10 μg/mL) or Hemin-MSC-EXO (10 μg/mL) and then exposed to a serum deprivation/hypoxia (SD/H) challenge (94% N_2_, 5% CO_2_, and 1% O_2_) for 72 h. After washing with PBS three times, NMCMs were fixed for 15 min and incubated with SA-β-gal staining solution at 37 °C without CO_2_ overnight. Finally, SA-β-gal positive cells, stained blue, were randomly imaged. The percentage of senescent NMCMs was calculated as the ratio of SA-β-gal positive NMCMs to total number of NMCMs obtained from five different fields of view.

### MitoTracker staining

The mitochondrial morphology of NMCMs was detected by MitoTracker Green FM (Invitrogen, M7514) according to the manufacturer’s protocol. Briefly, NMCMs were cultured in 24-well plates with cover slides and subjected to different treatments. After washing with PBS three times, they were incubated for 15 min at room temperature with DMEM supplemented with 20 nM MitoTracker Green FM. Finally, NMCMs were washed with PBS and imaged using a confocal microscope. Six fields were randomly observed and at least 300 cells per treatment group counted. The percentage of fragmented mitochondria among total number of cells was calculated.

### Transfection of miR-183-5p inhibitor and mimic

miR-control, miR-183-5p mimic and inhibitor were commercially acquired from GenePharma (Shanghai, China). Briefly, 1 × 10^6^ MSCs were plated on a 10-cm culture dish and transfected with 50 nM miR-183-5p mimic, inhibitor or miR-control using Lipofectamine 2000 transfection reagent (Invitrogen, 11668027) according to the manufacturer’s instructions. Subsequently, MSCs were cultured at 37 °C in a 5% CO_2_ incubator for 48 h and then harvested for further experiments.

### Luciferase assay

The 3′-UTR of human HMGB1 was inserted into the pGL3 luciferase reporter vector (Promega, Madison, WI, USA). Mutations in the seed region of the miR-183-5p-binding site in the HMGB1 3′-UTR were generated by PCR. 293T cells were seeded in 24-well plates and then co-transfected with the reporter plasmid (pGL3-HMGB1-3′-UTR or mutant HMGB1-3′-UTR vector) and miRNA control or miR-183-5p mimics using Lipofectamine 2000 (Invitrogen, 11668027). According to the manufacturer’s protocol, luciferase activity was determined 48 h after transfection using a Dual-Luciferase Reporter Assay System Kit (E1910, Promega).

### Real-time PCR

Total RNA from MSCs or MSC-EXO was isolated with TRIzol reagent (Takara, 2270A) and reverse transcription performed using a PrimeScript RT Reagent Kit (Takara, RR037A). RT-PCR of miR-183-5p and HMGB1 was performed using a One-Step TB Green^®^ PrimeScript™ RT-PCR Kit (Takara, RR820A). The mouse HMGB1 primer was: F: 5′-GCTGACAAGGCTCGTTATGAA-3′, R: 5′-CCTTTGATTTTGGGGCGGTA-3′. GAPDH and U6 were used as the internal reference. The miR-183-5p and U6 primers were obtained from GenePharma. The expression of miR-183-5p was normalized to the expression of U6 using the 2^−ΔΔCt^ cycle threshold method.

### Western blotting

Total protein of differently treated NMCMs and heart tissue from different experimental groups were extracted using a total protein extraction kit (Bestbio, BB-3101). After measuring the concentration using a BCA assay kit (Thermo, 231227), 30 μg protein was resolved by 10% Tris–glycine gel electrophoresis and then transferred onto a PVDF membrane. Subsequently, the membrane was blocked by 5% fat-free milk in TBST and incubated overnight at 4 °C with the following antibodies: anti-CD63 (Abcam, ab134045), anti-CD81 (Abcam, ab109201), anti-TSG101 (Abcam, ab125011), anti-Alix (Abcam, ab186429), anit-Calnexin (Proteintech, 10427-2-AP), anti-p21 (Abcam, ab109199), anti-p53 (Abcam, ab26), anti-p-Drp1 (Ser616) (CST, 3455), anti-Drp1 (CST, 14647), anti-Mfn2 (Abcam, ab124773), anti-Mfn1 (Abcam, ab57602), anti-p-ERK (CST, 9101), anti-ERK (CST, 4695), anti-HMGB1 (Abcam, ab18256) and anti-GAPDH (CST, 2118). Next, after washing three times with TBST, the PVDF membrane was incubated with secondary antibodies (1:1000, CST) at room temperature for 1 h and exposed in a dark room. The quantification of western blotting was analyzed with GAPDH as the internal reference using Image J software (National Institutes of Health, Bethesda, MD, USA) in three independent experiments.

### Exosomal miRNA sequencing

Total RNA was extracted from MSC-EXO and Hemin-MSC-EXO using a miRNeasy^®^ Mini kit (Qiagen, 217004). Degradation and contamination of RNA were assessed and the concentration and purity of RNA measured. After cutting into 18-30nt, small RNAs were reverse-transcribed to cDNA and a cDNA library generated. Gene Denovo Biotechnology Co. (Guangzhou, China) sequenced cDNA using Illumina HiSeqTM 2500. Raw reads were further filtered, then microRNA aligned and identified. miRNA expression profiles, miRNA Principal Component, miRNA Expression Pattern Clustering, differentially expressed miRNA (DE miRNA), Target gene Prediction and Target gene functional enrichment were analyzed. DE miRNA was identified through fold change > 1.5 and Q value < 0.001 with the threshold set for up- and down-regulated genes.

### MI model and transplantation of MSC-EXO

All animal experiments were approved by the Committee on the Use of Live Animals in Teaching and Research (CULTAR) of the Guangdong Provincial People’s Hospital for Laboratory Animal Medicine (No. KY-Z-2020-486-02). Male C57BL/6J mice (6–8 weeks, 20–25 g) were used for an acute MI model induced by ligation of the left anterior descending coronary artery (LAD) using an 8–0 nylon suture as previously described. After LAD ligation, mice were randomly assigned to one of the following treatments: (1) phosphate-buffered saline (PBS) (MI group, n = 12); (2) 20 μg MSC-EXO (MSC-EXO, n = 11); (3) 20 μg Hemin-MSC-EXO (Hemin-MSC-EXO group, n = 12); (4) 20 μg miR-183-5p^KD^-Hemin-MSC-EXO (miR-183-5p^KD^-Hemin-MSC-EXO, n = 12). All MSC-EXO were suspended in 30 μL PBS and intramuscularly injected 30 min after surgery into three sites at the border zone of the infarcted mouse heart. Another group of mice underwent thoracotomy without LAD ligation and served as the sham group (n = 6). Cardiac function in each mouse was assessed by transthoracic echocardiography (Ultramark 9; Soma Technology, Bloomfield, CT, USA) at baseline (before MI), and 1 and 28 days following MI. Left ventricle ejection fraction (LVEF) and left ventricle fraction shortening (LVFS) were calculated.

### Masson’s Trichrome staining

After measuring heart function at 28 days post-MI, all mice were sacrificed and the hearts quickly harvested. After washing with PBS three times, hearts were fixed, embedded and sectioned into 5 µm slices. Masson’s Trichrome staining was performed on heart sections from different groups. Images from 6 mice for each group were captured by scanning electron microscope (SU8010, Japan) and analyzed using Image-Pro Plus software (Media Cybernetics, Rockville, MD, USA). The percentage infarct size was determined as the sum of infarcted area from all sections/the sum of LV area from all sections × 100%.

### TEM assay

Mice heart tissue was harvested from the different experimental groups and fixed in 2% glutaraldehyde for 24 h. Samples were washed three times with cold 0.1 M phosphate buffer and fixed in phosphate acid buffer supplemented with 1% osmic acid for 2 h at room temperature. After dehydration with a gradient ethanol solution, samples were infiltrated with acetone-epoxy resin, embedded in epoxy resin and finally placed in an oven at 70 °C to polymerize. Embedded samples were sectioned into ultrathin sections (100 nm thickness) using a Leica EM UC7 microtome. Subsequently, sections were stained with 5% uranyl acetate for 10 min and Reynold’s lead citrate for 5 min. Finally, five randomly selected areas of mitochondria on each slide were captured using a 40–120 kV transmission electron microscope (Hitachi H600 Electron Microscope, Hitachi, Japan). Mitochondrial size (μm^2^) was analyzed with Image-Pro Plus software with measurements taken of at least 1000 mitochondria from six mice hearts in each group. Size < 0.6 μm^2^ was classified as mitochondria undergoing fragmentation.

### Immunohistochemistry

To determine cardiomyocyte senescence in the heart tissue from different groups, heart sections were immunohistochemically stained with anti-Troponin (1:100; Abcam, ab209809) and anti-p21 (1:100; Abcam, ab109199). To determine the blood vessel density, the heart sections were immunohistochemically stained with anti-CD31 (1:100; Abcam; ab19898). Five randomly selected areas on each slide were photographed under a fluorescence microscope (n = 6 per group). The percentage of Troponin/p21 double-positive cells was calculated per DAPI positive cells. The capillary density was expressed as the average number of CD31-positive blood vessels per field.

### Statistical analysis

Data are expressed as mean ± SD. Statistical analyses were performed using Prism 5.04 Software (GraphPad Software for Windows, San Diego, CA, USA). Comparison between two groups was analyzed by unpaired Student’s t-test and between multiple groups by one-way ANOVA followed by the Bonferroni test. A p value < 0.05 was considered statistically significant.

## Results

### Characterization of Hemin-MSC-EXO

**Fig. 1:**
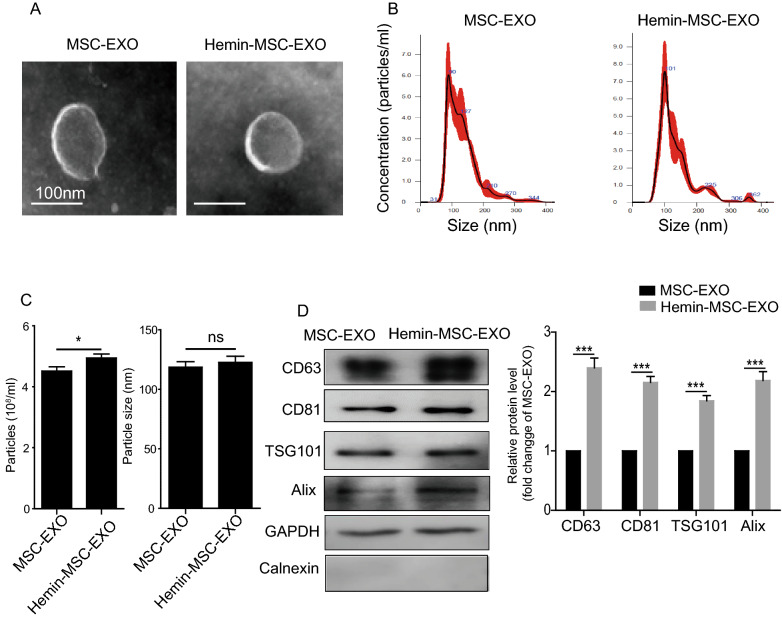
Characterization of MSC-EXO and Hemin-MSC-EXO. **A** Representative TEM images showing cup-shaped morphology of MSC-EXO and Hemin-MSC-EXO. **B** Particle size distribution of MSC-EXO and Hemin-MSC-EXO was measured by NTA. **C** The mean particle size and concentration of MSC-EXO and Hemin-MSC-EXO was analyzed by NanoSight NS300. **D** Representative images of Western blotting showing the expression of exosomal protein markers including Alix, TSG101, CD63, CD81, and endoplasmic reticulum membrane marker Calnexin in MSC-EXO and Hemin-MSC-EXO. n = 3 biological replicates for each group. Data are expressed as mean ± SD. *p < 0.05, ***p < *0.001.* ns = not significant

MSC-EXO and Hemin-MSC-EXO were isolated from their conditioned medium. TEM imaging analysis demonstrated that both MSC-EXO and Hemin-MSC-EXO displayed a typical cup-shaped morphology with a double-layer membrane structure (Fig. 1A). NTA was performed to examine the size distribution of MSC-EXO and Hemin-MSC-EXO (Fig. 1B). Both MSC-EXO and Hemin-MSC-EXO had a similar average diameter of 50–150 nm (Fig. 1C). Compared with MSC-EXO, the concentration of particles was significantly increased in Hemin-MSC-EXO (Fig. 1C). Western blotting demonstrated that both MSC-EXO and Hemin-MSC-EXO were positive for the known exosomal molecular markers, including CD63, CD81, TSG101 and Alix (Fig. 1D). GAPDH was used as an internal reference. Additionally, neither MSC-EXO nor Hemin-MSC-EXO expressed Calnexin, an endoplasmic reticulum membrane marker expressed in cells but not in EXO. Notably, the level of CD63, CD81, TSG101 and Alix was much higher in Hemin-MSC-EXO than MSC-EXO (Fig. 1D). These data showed that hemin pretreatment significantly enhanced MSC-EXO production as evidenced by increased particle concentration and level of classic EXO markers, but did not markedly modulate the size of the EXO. Collectively, these results confirmed the successful isolation of MSC-EXO and Hemin-MSC-EXO.

### Intramyocardial injection of Hemin-MSC-EXO improves cardiac function following infarction in mice

**Fig. 2:**
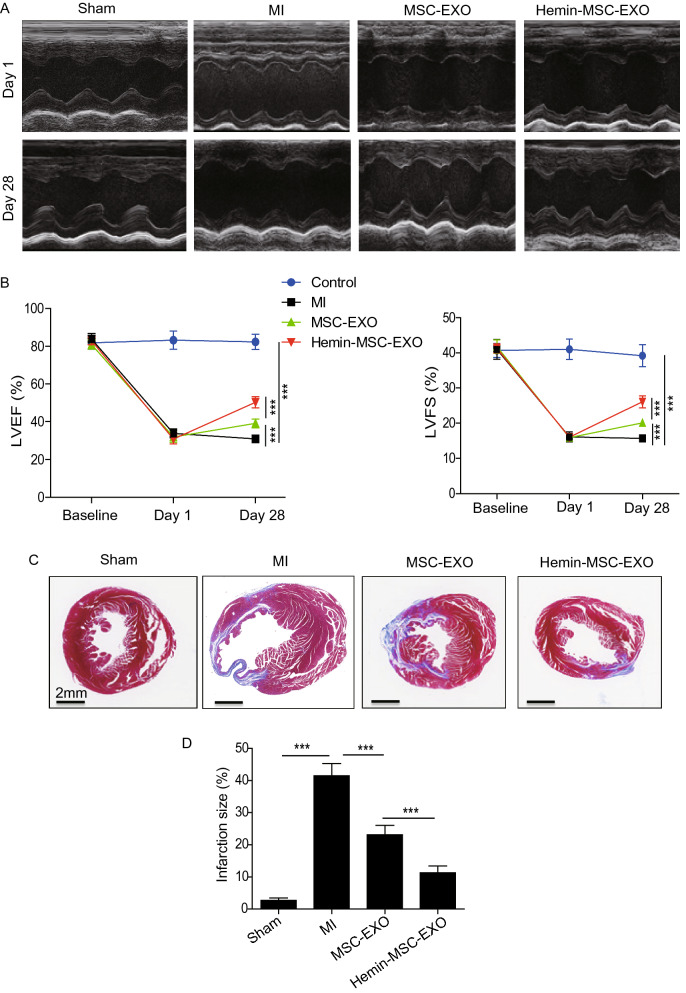
Hemin-MSC-EXO treatment improved cardiac function in mice following infarction. **A** Representative echocardiographic images were taken 1 day and 28 days after MI in mice that received PBS, MSC-EXO or Hemin-MSC-EXO treatment or sham mice. **B** The LVEF and LVFS were calculated at baseline (before MI), 1 and 28 days in sham or mice with MI that received PBS, MSC-EXO or Hemin-MSC-EXO treatment. **C** Representative images of Masson’s trichrome staining of heart sections from sham or mice with MI that received PBS, MSC-EXO or Hemin-MSC-EXO treatment. Red, myocardium; blue, scarred fibrosis. **D** Quantitative analysis of infarct size in control or mice with MI that received injections of PBS, MSC-EXO or Hemin-MSC-EXO treatment. Data are expressed as mean ± SD. n = 6 mice for each group, ***p < *0.001*

To assess the cardioprotective effects of Hemin-MSC-EXO, we intramyocardially injected Hemin-MSC-EXO into mice with MI. The heart function of mice from different groups was measured by echocardiography at baseline (Day 0), and 1 and 28 days post-MI. Representative images of echocardiography were taken at 1 day and 28 days post-MI (Fig. 2A). Echocardiography revealed that compared with the sham group, LVEF and LVFS were dramatically reduced on day 1 in all surgery groups, suggesting that the mouse MI model was successfully established (Fig. 2A, B). Furthermore, LVEF and LVFS at day 1 was similar among the MI, MSC-EXO and Hemin-MSC-EXO groups, suggesting a comparable degree of MI (Fig. 2A, B). As shown in Fig. 2B, compared with the MI group, LVEF and LVFS were greatly increased in all EXO-treated groups at 28 days post injection (Fig. 2B). Furthermore, LVEF and LVFS were much higher in the Hemin-MSC-EXO group compared with the MSC-EXO group, indicating that Hemin-MSC-EXO was superior to MSC-EXO in improving heart function following infarction (Fig. 2B). Similarly, Masson’s trichrome staining analysis showed that the infarcted size was significantly reduced in the MSC-EXO group and Hemin-MSC-EXO group compared with the MI group. Notably, Hemin-MSC-EXO treatment showed a better effect in attenuating heart infarction than MSC-EXO (Fig. 2C, D). To further assess the cardioprotective effects of MSC-EXO, we examined the capillary density using CD31 staining in mice hearts from the different groups at 28 days post-transplantation. As shown in Additional file 1: Figure S1, the capillary density was significantly increased in the MSC-EXO group and Hemin-MSC-EXO compared with the MI group. Furthermore, the capillary density was much higher in Hemin-MSC-EXO group than the MSC-EXO group (Additional file 1: Figure S1). Collectively, these findings suggest that MSC-EXO provided cardioprotective effects and improved heart function following MI. Importantly, the effect was superior for Hemin-MSC-EXO compared with MSC-EXO.

### Hemin-MSC-EXO ameliorate cardiomyocyte senescence and inhibit mitochondrial fission in infarcted hearts of mice

**Fig. 3:**
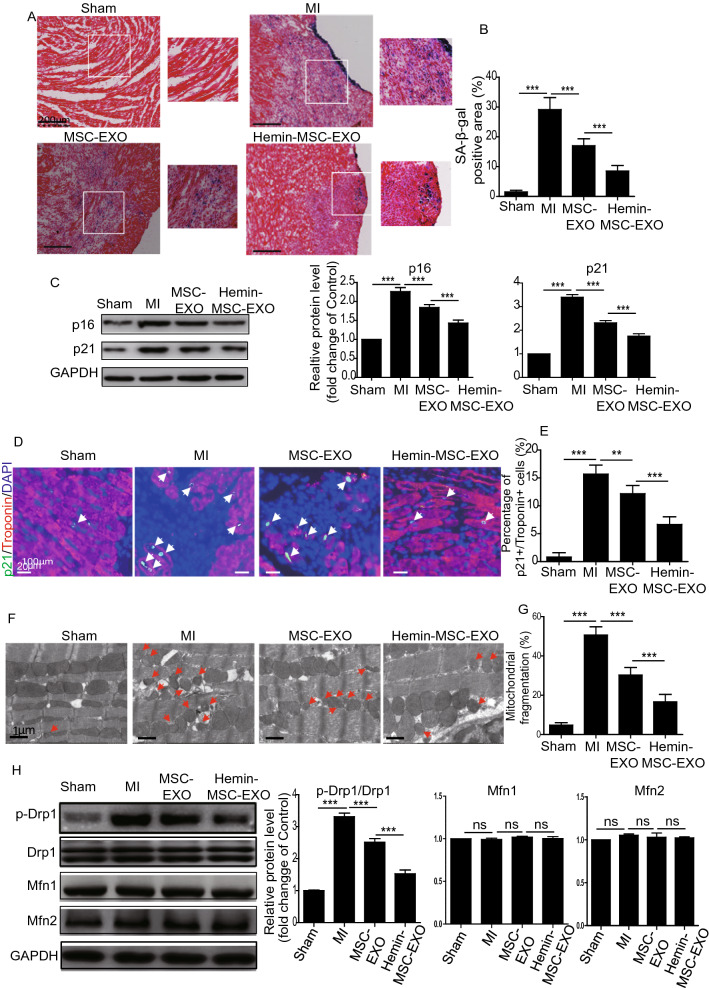
Hemin-MSC-EXO treatment ameliorated cardiomyocyte senescence and inhibited mitochondrial fission in infarcted hearts of mice. **A** Representative images of SA-β-gal staining at 28 days post infarction in sham and mice with MI that received PBS, MSC-EXO or Hemin-MSC-EXO treatment. **B** Quantitative analysis of SA-β-gal positive area in control or mice with MI that received PBS, MSC-EXO or Hemin-MSC-EXO treatment. **C** Western blotting and quantitative analysis of the expression level of p16 and p21 in sham or mice with MI that received PBS, MSC-EXO or Hemin-MSC-EXO treatment. **D** Representative images of Troponin (red) and p21 (green) staining in sham or mice with MI that received PBS, MSC-EXO or Hemin-MSC-EXO treatment. **E** Quantitative analysis of Troponin^+^/p21^+^ double-positive cells in sham or mice with MI that received PBS, MSC-EXO or Hemin-MSC-EXO treatment. **F** Representative TEM images of mitochondria in the heart tissue from sham or mice with MI that received PBS, MSC-EXO or Hemin-MSC-EXO treatment. **G** Quantitative analysis of mitochondrial fragmentation in heart tissue from sham or mice with MI that received PBS, MSC-EXO or Hemin-MSC-EXO treatment. **H** Western blotting and quantitative analysis of the expression level of p-Drp1, Drp1, Mfn1 and Mfn2 in heart tissue from sham or mice with MI that received PBS, MSC-EXO or Hemin-MSC-EXO treatment. Data are expressed as mean ± SD. n = 6 mice for each group, **p < 0.01, ***p < *0.001,* ns = not significant

Previous study has shown that cardiomyocyte senescence plays a critical role in regulating heart dysfunction following infarction [28]. We assessed the protective effects of MSC-EXO and Hemin-MSC-EXO on cardiomyocyte senescence in the infarcted heart in mice. Beta-galactosidase (β-gal), a eukaryotic hydrolase localized in the lysosome, is active at an optimal pH (6.0) in senescent cells but not in proliferating cells. We performed senescence-associated β-galactosidase (SA-β-gal) staining to detect cardiomyocyte senescence. Compared with the sham group, SA-β-gal activity was significantly increased in the infarct and the peri-infarct area in the MI group but greatly reduced in the MSC-EXO and Hemin-MSC-EXO group. The latter demonstrated superior benefit to the MSC-EXO group (Fig. 3A, B). Western blotting also showed that the level of cellular senescence markers p16 and p21 was reduced in the EXO-treated groups compared with the MI group (Fig. 3C). To further verify whether MSC-EXO treatment ameliorated cardiomyocyte senescence, we performed cardiomyocyte marker Troponin and senescent marker p21 double staining. As shown in Fig. 3D, compared with the MI group, the percentage of Troponin ^+^ /p21 ^+^ double-positive cells was greatly reduced in the MSC-EXO and Hemin-MSC-EXO groups. Furthermore, compared with the MSC-EXO group, the injection of Hemin-MSC-EXO was more effective in attenuating cardiomyocyte senescence (Fig. 3D, E). Next, we examined the mitochondrial dynamics among the different groups. TEM analysis showed that mitochondrial fragmentation, as evidenced by smaller and more rounded mitochondria, was greatly increased in the MI group compared with the sham group, indicating enhanced mitochondrial fission (Fig. 3F, G). This damaging effect was greatly attenuated in the MSC-EXO group and Hemin-MSC-EXO group, with the latter showing a better protective effect (Fig. 3F, G). Western blotting also showed that compared with the sham group, the protein level of p-Drp1/Drp1 was significantly increased in the MI group but downregulated in the EXO-treated groups (Fig. 3H). Notably, there was no difference in Mfn1 or Mfn2 expression among the different groups (Fig. 3H). These data showed that Hemin-MSC-EXO ameliorated cardiomyocyte senescence and inhibited mitochondrial fission in infarcted hearts, indicating a possible relationship between cardiomyocyte senescence and mitochondrial fission.

### Hemin-MSC-EXO ameliorates cardiomyocyte senescence induced by SD/H challenge via inhibition of mitochondrial fission

**Fig. 4:**
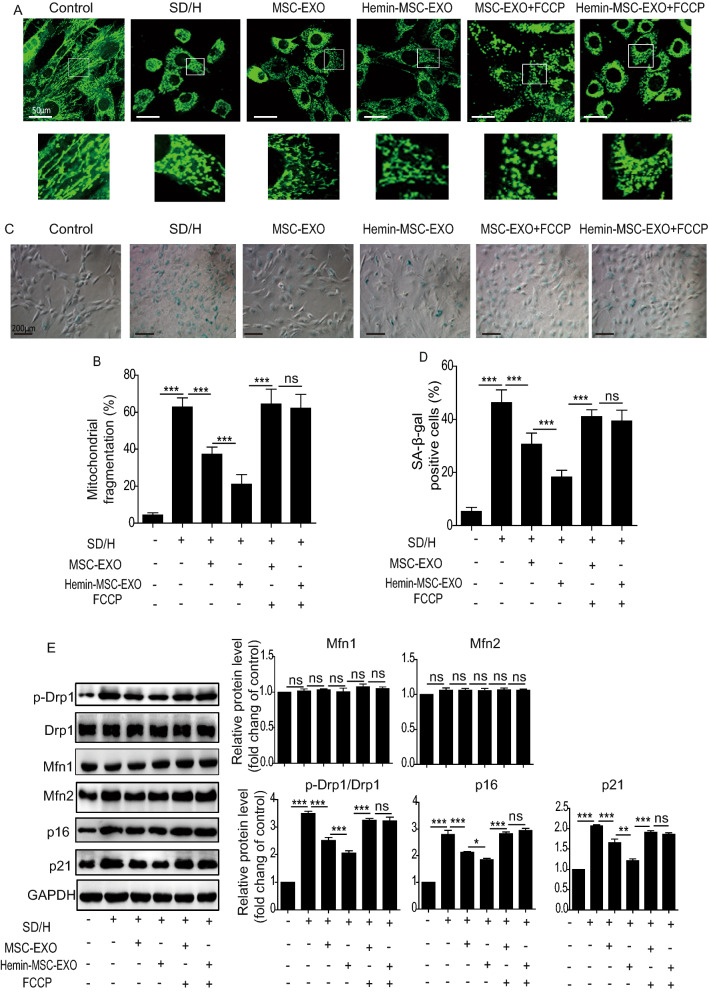
Hemin-MSC-EXO ameliorates cardiomyocyte senescence induced by SD/H challenge via inhibition of mitochondrial fission. **A** Representative images of the fragmented mitochondria in control, SD/H, SD/H + MSC-EXO, SD/H + Hemin-MSC-EXO, SD/H + MSC-EXO + FCCP, and SD/H + Hemin-MSC-EXO + FCCP-treated NMCMs. **B** Quantitative analysis of fragmented mitochondria in control, SD/H, SD/H + MSC-EXO, SD/H + Hemin-MSC-EXO, SD/H + MSC-EXO + FCCP, and SD/H + Hemin-MSC-EXO + FCCP-treated NMCMs. **C** Representative images of SA-β-gal staining in control, SD/H, SD/H + MSC-EXO, SD/H + Hemin-MSC-EXO, SD/H + MSC-EXO + FCCP, and SD/H + Hemin-MSC-EXO + FCCP-treated NMCMs. **D** Quantitative analysis of SA-β-Gal positive cells in control, SD/H, SD/H + MSC-EXO, SD/H + Hemin-MSC-EXO, SD/H + MSC-EXO + FCCP, and SD/H + Hemin-MSC-EXO + FCCP-treated NMCMs. **E** Western blotting and quantitative analysis of the expression level of p16, p21, p-Drp1, Drp1, Mfn1 and Mfn2 in control, SD/H, SD/H + MSC-EXO, SD/H + Hemin-MSC-EXO, SD/H + MSC-EXO + FCCP, and SD/H + Hemin-MSC-EXO + FCCP-treated NMCMs. SD/H means serum deprivation/hypoxia. n = 3 biological replicates for each group. Data are expressed as mean ± SD. *p < 0.05, **p < 0.01, ***p < *0.001,* ns = not significant

Based on the in vivo findings, we assessed the effects of Hemin-MSC-EXO on NMCM senescence induced by SD/H in vitro. As shown in Additional file 1: Figure S2, SD/H induced NMCM senescence at 72 h but the effect plateaued at 96 h (Additional file 1: Figure S2). Based on these results, SD/H-treated NMCM for 72 h were chosen for further study. To determine whether NMCMs could take up MSC-EXO, Dil-labeled MSC-EXO were incubated with NMCMs for 24 h. Confocal images showed that NMCMs could uptake labeled MSC-EXO (Additional file 1: Figure S3). Next, NMCMs were cultured under SD/H challenge and treated for 72 h with MSC-EXO or Hemin-MSC-EXO. SD/H challenge enhanced mitochondrial fragmentation (Fig. 4A, B) and increased SA-β-gal activity (Fig. 4C, D) and the protein level of p16, p21 and p-Drp1/Drp1 (Fig. 4E) in NMCM. MSC-EXO and Hemin-MSC-EXO treatment greatly downregulated the increased mitochondrial fragmentation, SA-β-gal activity, and p16, p21 and p-Drp1/Drp1 level induced by SD/H (Fig. 4A–E). Moreover, Hemin-MSC-EXO exhibited a better protective effect than MSC-EXO (Fig. 4A–E). Nonetheless these protective effects of MSC-EXO and Hemin-MSC-EXO were largely abrogated by mitochondrial fission activator FCCP (Fig. 4A–E). Collectively, these observations strongly suggest that Hemin-MSC-EXO exert their protective effects against cardiomyocyte senescence via regulation of mitochondrial fission.

### MiR-183-5p is enriched in Hemin-MSC-EXO and has protective effects

**Fig. 5:**
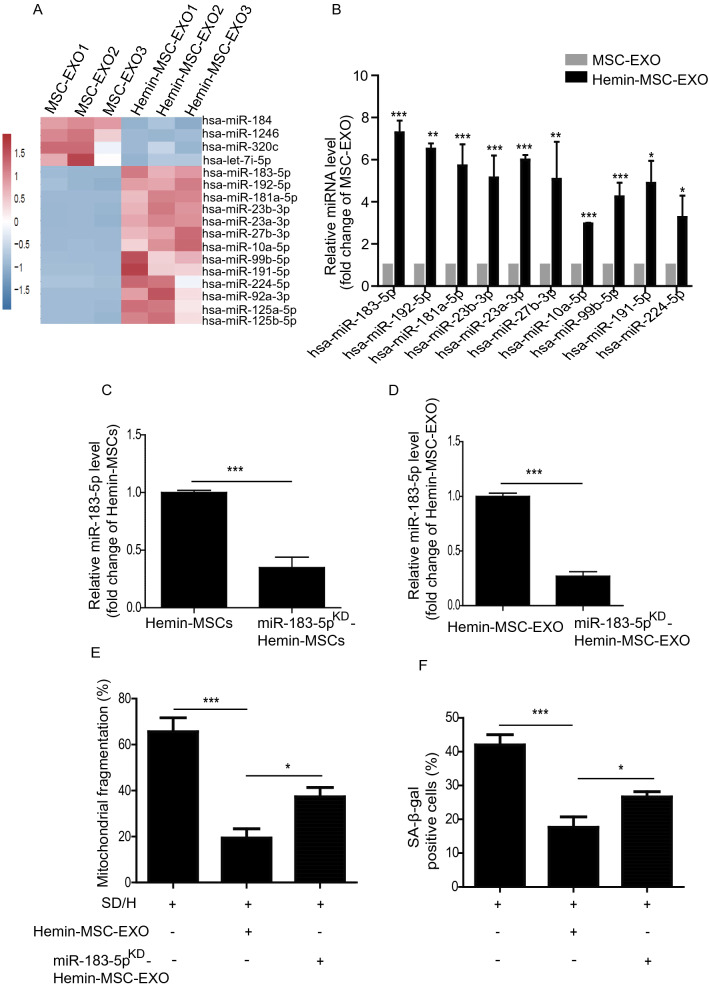
MiR-183-5p is enriched in Hemin-MSC-EXO and has protective effects on SD/H-induced NMCM injury. **A** RNA-seq analysis showed the differential expression of miRNAs between MSC-EXO and Hemin-MSC-EXO. **B** The top 10 highly-expressed miRNAs in Hemin-MSC-EXO were validated by qPCR. **C** Quantification of miR-183-5p expression in Hemin-pretreated MSCs and miR-183-5p^KD^-Hemin-MSC. **D** Quantification of miR-183-5p expression in Hemin-MSC-EXO and miR-183-5p^KD^-Hemin-MSC-EXO. **E** Quantitative analysis of mitochondrial fragmentation in NMCM treated with Hemin-MSC-EXO and miR-183-5p^KD^-Hemin-MSC-EXO under SD/H challenge. **F** Quantitative analysis of SA-β-Gal staining in NMCM treated with Hemin-MSC-EXO and miR-183-5p^KD^-Hemin-MSC-EXO under SD/H challenge. SD/H means serum deprivation/hypoxia. n = 3 biological replicates for each group. Data are expressed as mean ± SD. *p < 0.05, **p < 0.01, ***p < *0.001*

EXO are known to mediate their biological effect by delivering miRNAs. We performed RNA-seq of MSC-EXO and Hemin-MSC-EXO to identify the potential miRNA effectors. RNA-seq analysis revealed the differentially expressed miRNAs between MSC-EXO and Hemin-MSC-EXO (Fig. 5A). The top 10 highly-expressed miRNAs in Hemin-MSC-EXO were validated by qPCR. miR-183-5p was the most significantly increased miRNA in Hemin-MSC-EXO compared with MSC-EXO (Fig. 5B). Previous studies have shown that miR-183-5p plays an essential regulatory role in cardiovascular diseases [29, 30] and may regulate the beneficial effects in Hemin-MSC-EXO. Loss-of-function study was performed to examine the effects of exosomal miR-183-5p in MSC-EXO on NMCM senescence induced by the SD/H challenge. Expression of miR-183-5p was knocked down using miR-183-5p inhibitor in Hemin-pretreated MSCs and negative miR-control administered (Fig. 5C). Subsequently, EXO were isolated from the conditioned medium. As shown in Fig. 5D, expression of miR-183-5p was significantly reduced in EXO isolated from miR-183-5p inhibitor and Hemin-pretreated MSCs (miR-183-5p^KD^-Hemin-MSC-EXO) compared with Hemin-MSC-EXO (Fig. 5D). The effects of miR-183-5p^KD^-Hemin-MSC-EXO on SD/H-induced NMCM senescence were then explored. The increased mitochondrial fragmentation and cellular senescence of NMCM induced by SD/H were reduced by Hemin-MSC-EXO treatment, and the effect partially abrogated by miR-183-5p^KD^-Hemin-MSC-EXO (Fig. 5E, F). These data indicate that Hemin-MSC-EXO prevented NMCM senescence induced by SD/H and the beneficial effect was dependent on miR-183-5p.

### Exosomal miR-183-5p in Hemin-MSC-EXO inhibits mitochondrial fission via regulation of the HMGB1/ERK pathway

**Fig. 6:**
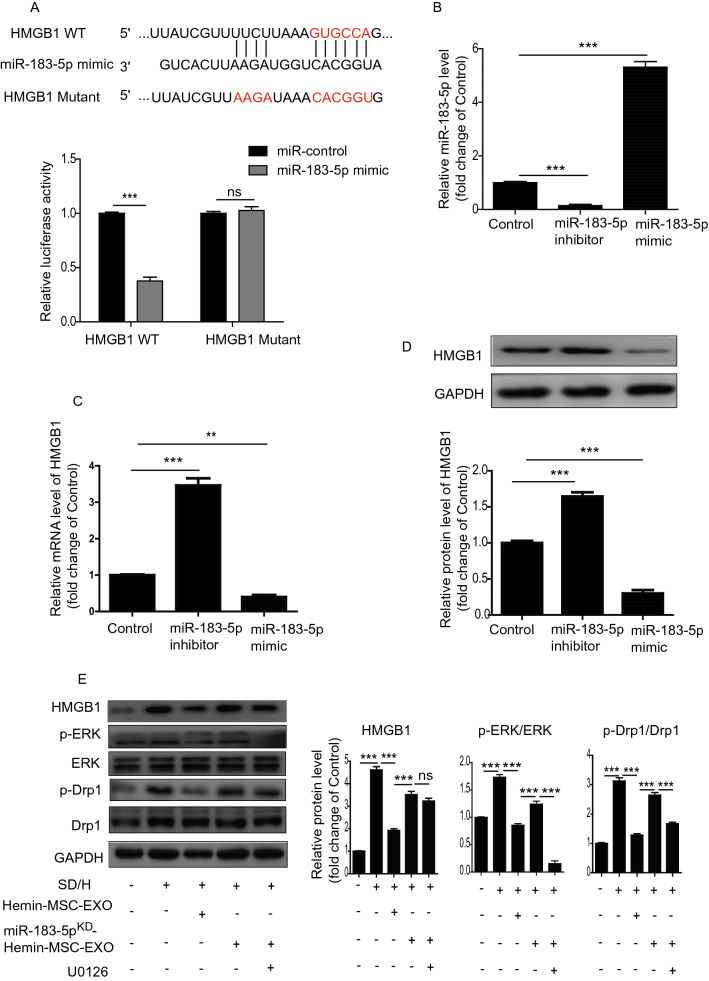
Exosomal miR-183-5p in Hemin-MSC-EXO inhibited mitochondrial fission via regulation of HMGB1. **A** Bioinformatics analysis predicted the binding sites between miR-183-5p and HMGB1, confirmed by dual-Luciferase reporter assay. **B** RT-PCR analysis of the level of miR-183-5p in control, miR-183-5p mimic and miR-183-5p inhibitor-treated NMCMs. **C** RT-PCR analysis of the level of HMGB1 in control, miR-183-5p mimic and miR-183-5p inhibitor-treated NMCMs. **D** Western blotting analysis of HMGB1 level in control, miR-183-5p mimic and miR-183-5p inhibitor-treated NMCMs. **E** Western blotting analysis of the level of HMGB1, p-Drp1, Drp1, p-ERK, ERK in control, SD/H, SD/H + Hemin-MSC-EXO, SD/H + miR-183-5p^KD^-Hemin-MSC-EXO and SD/H + miR-183-5p^KD^-Hemin-MSC-EXO + U0126 treated NMCMs. n = 3 biological replicates for each group. Data are expressed as mean ± SD. **p < 0.01, ***p < *0.001,* ns = not significant

Previous studies have shown that the HMGB1/ERK signaling pathway plays a critical role in regulating mitochondrial dynamics [31, 32]. We first examined the protein level of HMGB1, p-ERK in MI mice that received MSC-EXO and Hemin-MSC-EXO treatment. Compared with the sham group, expression of HMGB1, p-ERK was remarkably upregulated in the MI group but reduced in MSC-EXO and Hemin-MSC-EXO groups: the latter showed superior benefit to the MSC-EXO group (Additional file 1: Figure S4). Combined with the decreased mitochondrial fission in Hemin-MSC-EXO treated heart tissue (Fig. 3F–H), we presumed that Hemin-MSC-EXO inhibited mitochondrial fission via regulation of the HMGB1/ERK pathway. The binding sites between miR-183-5p and its potential target genes were predicted using Targetscan (http://www.targetscan.org/). We found that HMGB1 had a binding sequence with miR-183-5p at 3′-UTR, verified by luciferase reporter assay (Fig. 6A). Next, we used miR-183-5p mimic or inhibitor to manipulate the expression of miR-183-5p in NMCMs. The level of miR-183-5p was significantly decreased in miR-183-5p inhibitor-treated NMCMs but enhanced in miR-183-5p mimic-treated NMCMs (Fig. 6B). RT-PCR and Western blotting analysis showed that the expression of HMGB1 was enhanced by miR-183-5p inhibitor but downregulated by miR-183-5p mimic in NMCMs (Fig. 6C, D). Importantly, treatment with Hemin-MSC-EXO dramatically downregulated the expression of HMGB1, p-ERK/ERK and p-Drp1/Drp1 induced by SD/H in NMCMs (Fig. 6E). Nonetheless compared with Hemin-MSC-EXO treatment, knockdown of miR-183-5p in Hemin-MSC-EXO upregulated the expression of HMGB1, p-ERK/ERK, and p-Drp1/Drp1, and these effects were partially abrogated by ERK inhibitor U0126 (Fig. 6E). Collectively, in Hemin-MSC exosomal miR-183-5p inhibited mitochondrial fission induced by SD/H in NMCMs via regulation of the HMGB1/ERK pathway.

### Knockdown of miR-183-5p reduced Hemin-MSC-EXO-mediated cardioprotective effects

**Fig. 7:**
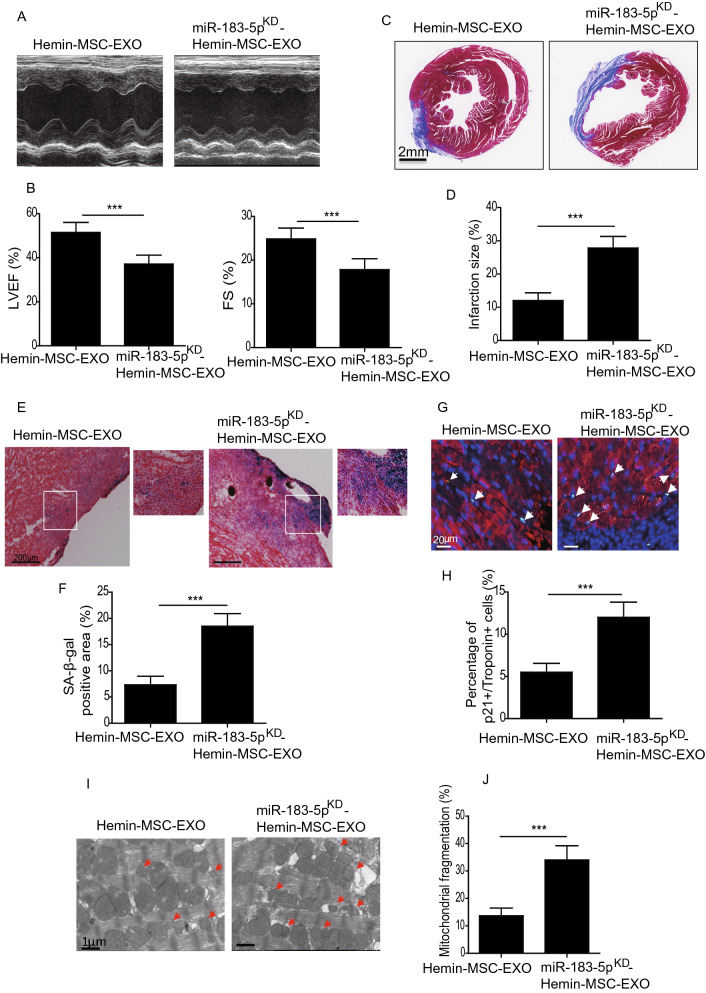
Downregulation of exosomal miR-183-5p reduced the cardioprotective effects of Hemin-MSC-EXO. **A** Representative echocardiographic images 28 days after MI in mice that received Hemin-MSC-EXO or miR-183-5p^KD^-Hemin-MSC-EXO treatment. **B** The LVEF and LVFS were calculated at 28 days in mice with MI that received Hemin-MSC-EXO or miR-183-5p^KD^-Hemin-MSC-EXO treatment. **C** Representative images of Masson’s trichrome staining of heart sections from mice with MI that received Hemin-MSC-EXO or miR-183-5p^KD^-Hemin-MSC-EXO treatment. Red, myocardium; blue, scarred fibrosis. **D** Quantitative analysis of infarct size in mice with MI that received Hemin-MSC-EXO or miR-183-5p^KD^-Hemin-MSC-EXO treatment. **E** Representative images of SA-β-gal staining of heart tissue from mice with MI that received Hemin-MSC-EXO or miR-183-5p^KD^-Hemin-MSC-EXO treatment. **F** Quantitative analysis of SA-β-gal positive area in heart tissue from mice with MI that received Hemin-MSC-EXO or miR-183-5p^KD^-Hemin-MSC-EXO treatment. **G** Representative images of Troponin (red) and p21 (green) staining in heart tissue from mice with MI that received Hemin-MSC-EXO or miR-183-5p^KD^-Hemin-MSC-EXO treatment. **H** Quantitative analysis of Troponin^+^/p21^+^ double-positive cells in heart tissue from mice with MI that received Hemin-MSC-EXO or miR-183-5p^KD^-Hemin-MSC-EXO treatment. **I** Representative TEM images of mitochondria in heart tissue from mice with MI that received Hemin-MSC-EXO or miR-183-5p^KD^-Hemin-MSC-EXO treatment. **J** Quantitative analysis of mitochondrial fragmentation in heart tissue from mice with MI that received Hemin-MSC-EXO or miR-183-5p^KD^-Hemin-MSC-EXO treatment. Data are expressed as mean ± SD. n = 6 mice for each group, ***p < *0.001*

To further validate the cardioprotective effects of exosomal miR-183-5p in Hemin-MSC-EXO, we tested the therapeutic efficacy of miR-183-5p^KD^-Hemin-MSC-EXO in a mouse MI model. At 28 days post MI, compared with the Hemin-MSC-EXO group, LVEF and LVFS were greatly reduced in the miR-183-5p^KD^-Hemin-MSC-EXO group (Fig. 7A, B). Furthermore, miR-183-5p^KD^-Hemin-MSC-EXO-treated mice had a significantly larger scarred area compared with Hemin-MSC-EXO-treated mice (Fig. 7C, D). Moreover, SA-β-gal activity was significantly enhanced in the infarct and the peri-infarct area of miR-183-5p^KD^-Hemin-MSC-EXO-treated heart tissue compared with the Hemin-MSC-EXO group (Fig. 7E, F). Similarly, the percentage of Troponin^+^/p21^+^ double-positive cells was greatly increased in the miR-183-5p^KD^-Hemin-MSC-EXO group compared with the Hemin-MSC-EXO group (Fig. 7G, H). Mitochondrial fragmentation in the miR-183-5p^KD^-Hemin-MSC-EXO group was much higher than in the Hemin-MSC-EXO group (Fig. 7I, J). These results showed that downregulation of miR-183-5p expression reduced the Hemin-MSC-EXO-mediated cardioprotective effects.

## Discussion

This study produced several major findings. First, ischemic injury induced cardiomyocyte senescence via activation of mitochondrial fission that further contributed to cardiac dysfunction. Second, MSC-EXO transplantation exhibited a cardioprotective effect in MI, and this effect was augmented by Hemin pretreatment. Third, Hemin-MSC-EXO improved cardiac function following infarction by attenuating cardiomyocyte senescence in mice. Finally, the beneficial effects of Hemin-MSC-EXO were partially mediated by miR-183-5p that ameliorated cardiomyocyte senescence in the infarcted heart via regulation of the HMGB/ERK signaling pathway.

Over the past decades, an increasing number of animal studies and clinical trials have shown that MSC-based therapy is a novel strategy for MI treatment due to their unique properties that include easy isolation, immunomodulatory properties and multiple differentiation capacity [33–36]. Further in-depth studies have revealed that the cardioprotective effects of MSCs are predominantly due to their paracrine factors, including growth factors, cytokines and EXO, rather than trans-differentiation ability. More importantly, compared with MSC transplantation, MSC-EXO have several advantages such as long-term stability, minimum risk of immunogenicity and no risk of tumorigenesis [37]. Indeed, MSC-EXO-based therapy, as a new cell-free therapy, has attracted considerable attention for various diseases including MI. MSC-EXO exert their cardioprotective effects by transferring endogenous molecules including lncRNA, miRNAs, and proteins to regulate apoptosis, cardiac angiogenesis and inflammation in the ischemic heart. Therefore, modifying MSC-EXO in vitro to alter their molecular components is a novel strategy to augment their cardioprotective effects. EXO derived from MSCs overexpressing macrophage migration inhibitory factor were superior to MSC-EXO in improving heart function following infarction in rats [38]. In addition to genetic modification, environmental stimuli are another novel strategy to optimize MSC-EXO. Our previous studies showed that Hemin pretreatment enhanced the therapeutic efficacy of MSCs for MI [24]. This prompted us to investigate whether Hemin pretreatment could affect the cardioprotective effects of MSC-EXO in MI. As expected, Hemin-MSC-EXO had superior cardioprotective effects in repair of the infarcted heart in mice.

Although the mechanisms have not been fully explored, it has been well documented that EXO mediates cardioprotective effects by delivering specific miRNA to injured cardiomyocytes. It has been reported that MSC-EXO attenuate cardiac dysfunction by transferring miR-25-3p to prevent cardiomyocyte apoptosis and inflammatory response following infarction [39]. Transplantation of MSCs has been shown to improve heart function following infarction via the exosomal transfer of miR-125b to reduce autophagic flux in infarcted hearts, and knockdown of miR-125b partially abrogated the cardioprotective effects of MSC-EXO [40]. Nonetheless the role of the miRNAs contained in MSC-EXO in cardiac repair has not been investigated. In the current study, the expression profile of miRNAs revealed that miR-183-5p was significantly elevated in Hemin-MSC-EXO compared with MSC-EXO. It has been reported that miR-183-5p protects against myocardial ischemia/reperfusion-induced heart injury by repressing voltage-dependent anion channel 1 (VDAC1) [41]. Importantly, knockdown of miR-183-5p in Hemin-MSC-EXO significantly downregulated their cardioprotective effects, suggesting that the protective effects of Hemin-MSC-EXO were at least partially mediated by miR-183-5p.

Mitochondria, the major source of ATP in cardiomyocytes, undergo fission and fusion to maintain their function. Disequilibrium of mitochondrial fission and fusion contributes to the development of various cardiovascular diseases, including cardiac hypertrophy and MI [42, 43]. Recently, abnormal mitochondrial dynamic-induced cardiomyocyte senescence has been reported to be involved in cardiac injury [15, 44, 45]. In this study, Hemin-MSC-EXO treatment significantly inhibited mitochondrial fission and senescence of cardiomyocytes induced by SD/H and in the ischemic heart tissue. Notably, these effects were partially abolished by the mitochondrial fission activator, suggesting that the cardioprotective effects of Hemin-MSC-EXO are paritially due to amelioration of mitochondrial fission-induced cardiomyocyte senescence. Furthermore, downregulation of miR-183-5p in Hemin-MSC-EXO reversed the anti-mitochondrial fission capacity, indicating that exosomal miR-183-5p plays an essential role in its regulation. Nonetheless the potential mechanisms underlying exosomal miR-183-5p regulation of mitochondrial fission remain unclear. HMGB1, a highly DNA-binding protein, promotes gene transcription. Importantly, HMGB1 can function as an extracellular signaling molecule under pathophysiological conditions to regulate a range of responses, including inflammation, immunity, and cell death [32]. Accumulating evidence has revealed that HMGB1 regulates mitochondrial dynamics under stress conditions via multiple signal pathways [46, 47]. It has been reported that HMGB1 induces mitochondrial fission via the ERK/Drp1 signaling pathway in colorectal cancer [32]. In the current study, HMGB1 and p-ERK were significantly increased in the ischemic heart tissue but decreased in the Hemin-MSC-EXO treatment group. Furthermore, we revealed that HMGB1 is a potential target of miR-183-5p that was greatly enriched in Hemin-MSC-EXO. Thus, our results provide evidence that exosomal miR-183-5p in Hemin-MSC-EXO inhibits SD/H-induced mitochondrial fission in cardiomyocytes via regulation of the HMGB1/ERK signaling pathway.

This study also has several limitations. First, in addition to miR-183-5p, whether additional bioactive contents that are enriched in the Hemin-MSC-EXO contribute to the cardioprotective effects requires further investigation. Second, whether other mechanisms, including telomere shortening and autophagy injury, mediate ischemia-induced cardiomyocyte senescence remains to be determined in addition to mitochondrial dysfunction. Third, the long-term impact of Hemin-MSC-EXO on heart function recovery following infarction was not examined in this study. Fourth, although our data demonstrate that hemin pretreatment can upregulate miR-183-5p in MSC-EXO, the signaling pathways between hemin and miR-183-5p need further elucidation.

In conclusion, our study demonstrated that Hemin-MSC-EXO were superior to MSC-EXO in improving heart function following infarction. MiR-183-5p enriched in Hemin-MSC-EXO, partially via regulation of the HMGB1/ERK pathway, inhibited ischemia-induced cardiomyocyte senescence to enhance the cardioprotective effects by regulating mitochondrial fission. Our study provides a novel candidate target to repair heart injury following infarction.

## Supplementary Information

**Additional file 1:** **Figure S1.** Transplantation of Hemin-MSC-EXO improves angiogenesis in mice hearts following infarction. **Figure S2.** SD/H-induced NMCM senescence in a time-dependent manner. **Figure S3.** Confocal images show that red fluorescence of Dil labeled MSC-EXO were endocytosed by NMCMs. **Figure S4.** Western blotting and quantitative analysis of the expression level of HMGB1 and p-ERK in heart tissue from sham or mice with MI that received PBS, MSC-EXO or Hemin-MSC-EXO treatment.

## Data Availability

All data and materials are available on request.
