# Supplementary material for: Hemin enhances the cardioprotective effects of mesenchymal stem cell-derived exosomes against infarction via amelioration of cardiomyocyte senescence
Source: J Nanobiotechnology. 2021 Oct 21;19:332. doi: 10.1186/s12951-021-01077-y (PMC8532335; doi:10.1186/s12951-021-01077-y)

**Figure S1. Transplantation of Hemin-MSC-EXO improves angiogenesis in mice hearts following infarction**

(A) Representative images of CD31 staining in the ischemic heart of mice at 4 weeks among the different groups. (B) Quantitative analysis of the capillary density in the ischemic heart at 4 weeks among the different groups. Data are expressed as mean ± SD. n = 6 mice for each group, ***p< 0.01, ***p< 0.001.*

**
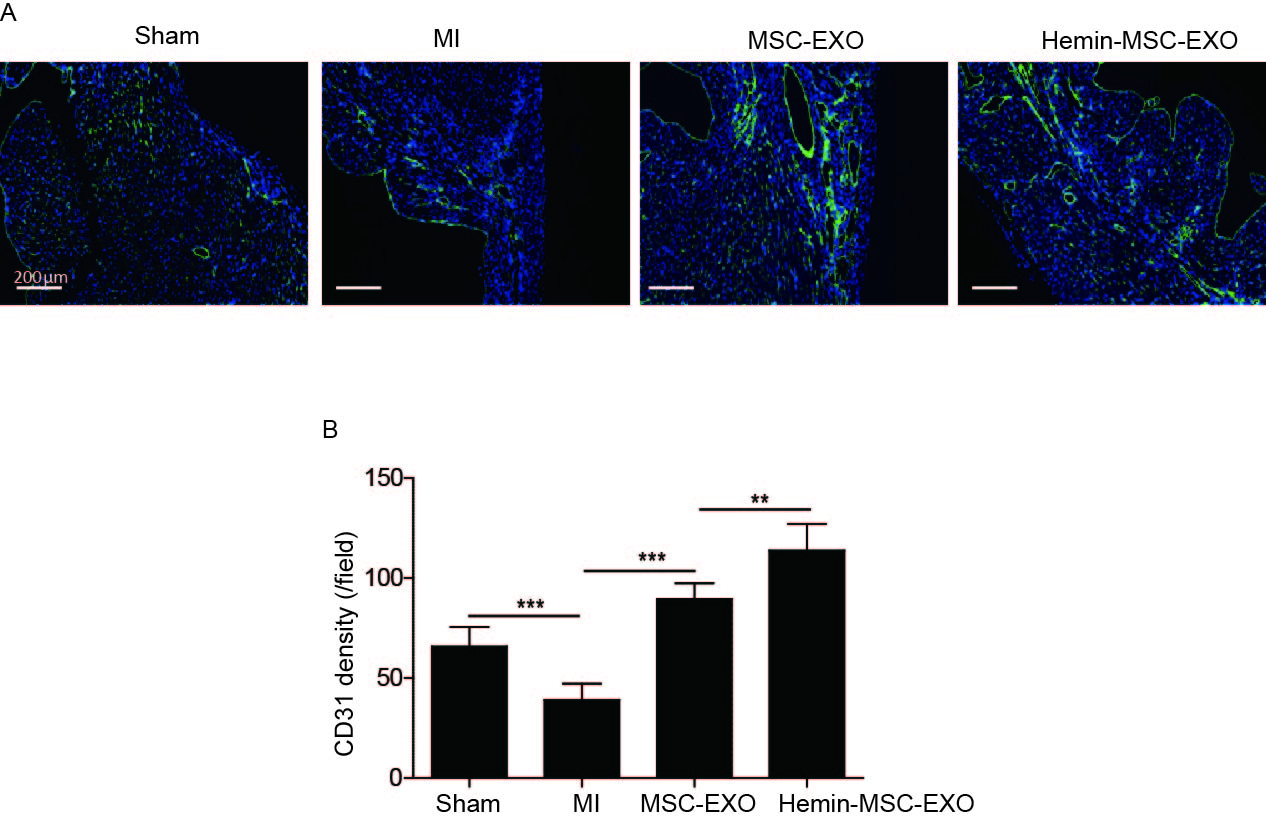
**

**Figure S2. SD/H-induced NMCM senescence in a time-dependent manner.**

(A) Representative images of SA-β-gal staining in NMCMs treated with SD/H for 0h, 24h, 48h, 72h and 96h. (B) Quantitative analysis of SA-β-gal positive cells in NMCMs subjected to SD/H for 0h, 24h, 48h, 72h and 96h. **p<0.05*. ****p<0.001*. ns=not significant.


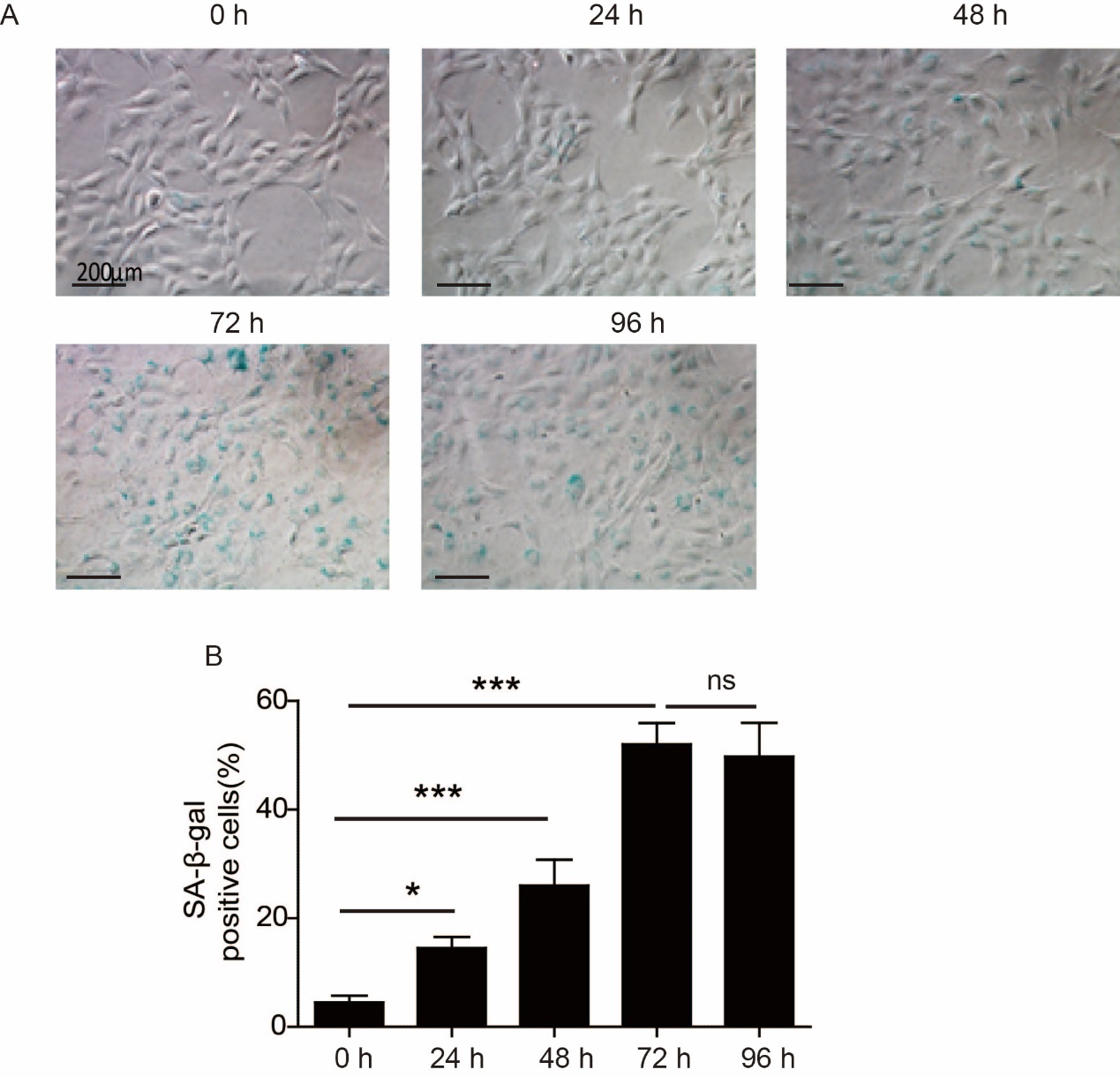


**Figure S3. Confocal images show that red fluorescence of Dil labeled MSC-EXO were endocytosed by NMCMs.**

**
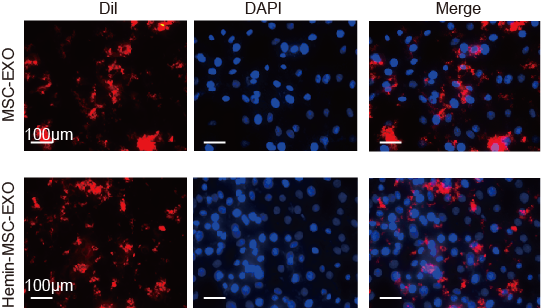
**

**Figure S4. Western blotting and quantitative analysis of the expression level of HMGB1 and p-ERK in heart tissue from sham or mice with MI that received PBS, MSC-EXO or Hemin-MSC-EXO treatment.** Data are expressed as mean±SD. n = 6 mice for each group*, ***p<0.001*.


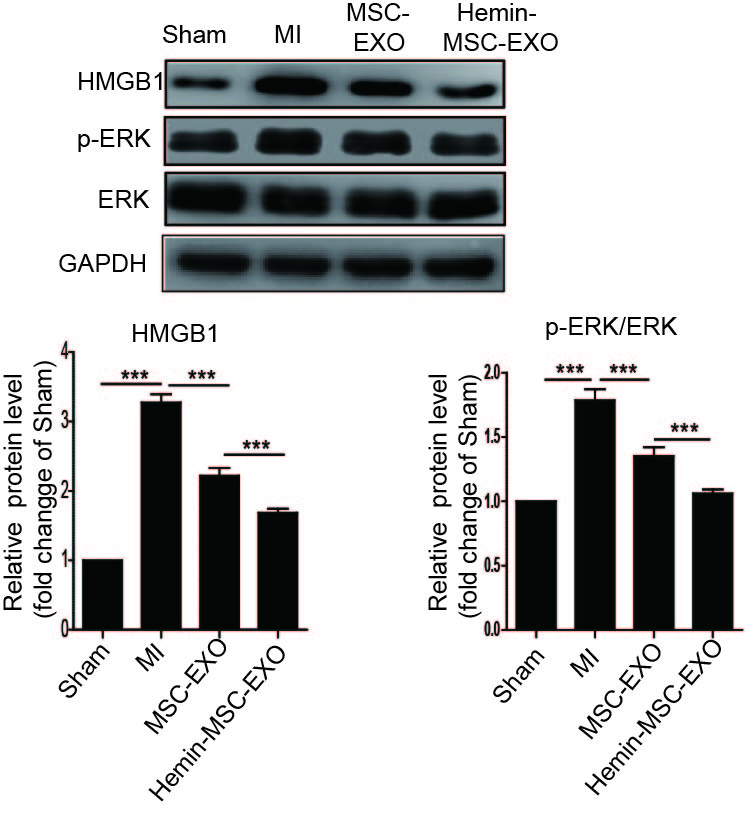

Supplement: Supplementary file 1 — Additional file 1: Figure S1. Transplantation of Hemin-MSC-EXO improves angiogenesis in mice hearts following infarction. Figure S2. SD/H-induced NMCM senescence in a time-dependent manner. Figure S3. Confocal images show that red fluorescence of Dil labeled MSC-EXO were endocytosed by NMCMs. Figure S4. Western blotting and quantitative analysis of the expression level of HMGB1 and p-ERK in heart tissue from sham or mice with MI that received PBS, MSC-EXO or Hemin-MSC-EXO treatment. [file 12951_2021_1077_MOESM1_ESM.docx]
